# Mechanism of Earthquake Simulation as a Prenatal Stressor Retarding Rat Offspring Development and Chinese Medicine Correcting the Retardation: Hormones and Gene-Expression Alteration

**DOI:** 10.1155/2012/670362

**Published:** 2012-11-26

**Authors:** X. G. Zhang, H. Zhang, R. Tan, J. C. Peng, X. L. Liang, Q. Liu, M. Q. Wang, X. P. Yu

**Affiliations:** ^1^The School of Nursing, Chengdu University of T.C.M., Chengdu 610075, China; ^2^School of Biology and Engineering, Southwest Jiaotong University, Chengdu 610031, China; ^3^Earthquake Emergency Security Center, Sichuan Provincial Seismological Bureau, Chengdu 610041, China; ^4^Molecular Laboratory of T.C.M., Chengdu University of T.C.M., Chengdu 610075, China; ^5^School of Humanity and Information Management, Chengdu Medical University, Chengdu 610075, China

## Abstract

We aimed to investigate the mechanism of shaking as a prenatal stressor impacting the development of the offspring and Chinese medicines correcting the alterations. Pregnant rats were randomized into earthquake simulation group (ESG), herbal group (HG) which received herbal supplements in feed after shaking, and control group (CG). Findings revealed body weight and open field test (OFT) score of ESG offspring were statistically inferior to the CG and HG offspring. The corticosterone levels of ESG were higher than those of CG but not than HG. The dopamine level of ESG was slightly lower than that of the CG and of HG was higher than that of ESG. The 5-HT of ESG was higher than CG and HG. The growth hormone level of the ESG was significantly lower than ESG but not than CG. Gene expression profile showed 81 genes upregulated and 39 genes downregulated in ESG versus CG, and 60 genes upregulated and 28 genes downregulated in ESG versus HG. Eighty-four genes were found differentially expressed in ESG versus CG comparison and were normalized in ESG versus HG. We conclude that maternal shaking negatively affected physical and nervous system development, with specific alterations in neurohormones and gene expression. Chinese herbal medicine reduced these negative outcomes.

## 1. Introduction

Maternal effects have been demonstrated as an essential factor for offspring development in many species. Because of the long period of perinatal mother-infant interaction in mammals, the growth and development and variations of offspring are very likely to be influenced by maternal impacts, leaving long-term consequences for both psychological and physiological health [1]. Recent human studies have shown that long-lasting and a wide variety of prenatal stressors, from anxiety and partner relationship problems to natural disasters, increase the risk for a diverse range of adverse neurodevelopmental outcomes in the child, including impaired cognitive development and behavioral problems [2, 3]. Animal experiments have convincingly demonstrated that prenatal maternal stress affects pregnancy outcome and results in early programming of brain functions with permanent changes in neuroendocrine regulation, gene expression, and behavior in offspring [4]. Prenatal restraint stress in rats is a common experimental model of early stress known to have long-term behavioral and neurobiological consequences [5, 6]. PS modifies the plastic responses of the adult brain, including the circuitry of the hippocampus-hypothalamus- pituitary-adrenalaxis (HHPA), that participate in the neuroendocrine control of feeding and metabolism in adult life [7].

As a typical prenatal stress, shaking can significantly impact the psychological and intellectual development of fetus and birth outcomes [8] in human. Naturally, earthquake is a fierce shaking. Tan et al. [9] reported that rates of birth defects after an earthquake were significantly higher than those before earthquake, whose spectrum was dramatically altered after earthquake, with the markedly increased occurrences of ear malformations; meanwhile the ratio of preterm birth after earthquake was significant increased than that of before earthquake. Oyarzo et al. [10] reported that women exposed to the February 27th 2010 Chilean earthquake during her first trimester delivered smaller newborns and they were more likely diagnosed with early preterm delivery, preterm delivery, and PROM but were less likely diagnosed with intrauterine growth retardation and late delivery compared to those exposed at third trimester, indicating disasters such as earthquakes are associated to adverse perinatal outcomes that impact negatively the entire maternal-neonatal healthcare system. Like the other alterations induced by PS in behavior those in learning and their direction appears to be dependent on the intensity, duration, and timing of the maternal stress [11].

In Chinese medicine, PS from shaking or an analog of earthquake is considered as a factor which impairs kidney Qi (*shen qi*) [12]. As kidney is the root of earlier heaven (the congenital constitution), it governs reproduction and development and holds oriffice of labor, whence agility and emanates. Jin Kui Shen Qi Wan (JKSQW) is a typical herbal formula supplementing kidney Qi, which recovers the physiological functions of kidney [13].

The current study involves shaking as a prenatal stressor. A first goal was to establish that earthquake simulation led to significant delays in development. A second goal was to examine whether Chinese traditional medicine could be used to address these negative effects. Based on the above information, we hypothesized parental kidney is injured from PS derived from earthquake simulation on rats, traits are handed down to offspring, showing development retardation; JKSQW could recover the dysfunctions of kidney whose underlying mechanism could involve development, hormones and gene expression alterations.

## 2. Materials and Methods

### 2.1. Grouping

Forty-five Sprague-Dawley (SD) female rats (230 g~270 g) and 45 male rats (225 g~261 g) were involved in this research. The rats were housed in a room with a temperature of 22°C, 12 hour light/dark cycle and fed with food and water *ad libitum*. After a week of adaption housing, the female rats were mated with the male rats. Pregnancy was confirmed by vaginal plug test. Then the 34 pregnant rats were randomized into three groups, control group (CG) (*n* = 11), earthquake simulation with conventional chow group (ESG) (*n* = 11), and earthquake plus herbal group (HG) (*n* = 12), and they were housed under pregnant rat cages until the delivery. With this procedure, all the groups were transferred with equivalent stress during pregnancy. There was no statistical difference of gestation time detected or body weight of the first day of gestation (CG: 234.87 ± 2.20, ESG: 234.98 ± 1.95, and HG: 235.16 ± 1.96, ANOVA test, *P* > 0.05 (g)) in the three groups. After delivery, all the litters of the three groups were housed with their mothers until the 25th day after birth. 

### 2.2. Earthquake Simulation

 The ESG cages housing pregnant rats were manually shaken up and down 3 times to simulate an initial earthquake and then were shaken for 50 timesover the next 15 minutes to modulate an aftershock [14]. The shaking was performed twice a day until delivery. Severity of the shake was measured with a seism velometer (DX-6Y2, Cheng Du Mei Huan Tech. Co. Ltd.), showing 9.6~10.5 of seismic intensity, 950 mg~1050 mg of vertical peak ground accelerations (PGA), which was similar to the PGA (1080 mg) of Wenchuan earthquake, May 12, 2008, China.

### 2.3. Chinese Herbal Formula Feed

The feed of HG rats was supplemented with herbal medicine until delivery, which consisted of (*Radix Rehmanniae Preparata* (Shu Di Huang), *Fructus Corni Officinalis* (Shan Zhu Yu), *Cortex Moutan Radicis* (Mu Dan Pi), *Rhizoma Dioscoreae Oppositae* (Shan Yao), *Sclerotium Poriae Cocos* (Fu Ling), *Rhizoma Alismatis Orientalis* (Ze Xie), *Radix Aconiti Lateralis Preparata* (Zhi Fu Zi), and *Cortex Cinnamomi Cassiae* (Rou Gui)) bought from Tong Ren Tang Technologies, Co., Ltd. The pill of *JKSQW *was grinded and added to the conventional feed 0.5~0.6 g/d.

### 2.4. Body Weight Measurement

Body weight (g) was measured at the 1st (day 0), 5th (day 5), 10th (day 10), 15th (day 15), 20th (day 20), and 25th (day 25) days after delivery in order to evaluate the body development of the offspring.

### 2.5. Open Field Test (OFT)

A square board (90 cm × 90 cm) painted with yellow and white squares (15 cm × 15 cm). The offspring of 25 days old was placed in the center of the board. We counted how many squares the offspring had crawled across in two minutes. One score was given only when the four paws of an offspring were in one square.

### 2.6. Hormone Assay

Thirty offspring were randomly selected from the groups, ten for each. Blood sample was taken from *arteria femoralis*. ELISA (R&D Systems China Co., Ltd.) was employed to determine the serum level of corticosterone (DZE 30590), dopamine (DZE 30238), 5-HT (DZE 30326), and growth hormone (DZE 30549).

### 2.7. Gene Expression Profile Chip Experiments

#### 2.7.1. RNA Extraction and Purification

Total RNA was extracted using TRIZOL Reagent (Cat no. 15596-018, technologies, Carlsbad, CA, US) following the manufacturer's instructions and checked for a RIN number to inspect RNA integration by an Agilent Bioanalyzer 2100 (Agilent technologies, Santa Clara, CA, US). Qualified total RNA was further purified by RNeasy mini kit (Cat no. 74106, QIAGEN, GmBH, Germany) and RNeasy micro kit (Cat no. 74004, QIAGEN, GmBH, Germany) and RNase-Free DNase Set (Cat no. 79254, QIAGEN, GmBH, Germany) (Table 1).

#### 2.7.2. RNA Amplification and Labeling

Total RNA was amplified and labeled by Low Input Quick Amp Labeling Kit, One-Color (Cat no. 5190-2305, Agilent technologies, Santa Clara, CA, US), following the manufacturer's instructions. Labeled cRNA were purified by RNeasy mini kit (Cat no. 74106, QIAGEN, GmBH, Germany).

#### 2.7.3. Hybridization

Each slide was hybridized with 1.65 *μ*g Cy3-labeled cRNA using Gene Expression Hybridization Kit (Cat no. 5188-5242, Agilent technologies, Santa Clara, CA, US) in Hybridization Oven (Cat no. G2545A, Agilent technologies, Santa Clara, CA, US), according to the manufacturer's instructions. After 17 hours hybridization, slides were washed in staining dishes (Cat no. 121, Thermo Shandon, Waltham, MA, US) with Gene Expression Wash Buffer Kit (Cat no. 5188-5327, Agilent technologies, Santa Clara, CA, US), following the manufacturer's instructions.

#### 2.7.4. Data Acquisition

Slides were scanned by Agilent Microarray Scanner (Cat no. G2565CA, Agilent technologies, Santa Clara, CA, US) with default settings: dye channel: Green, Scan resolution = 5 *μ*m, PMT 100%, 10%, 16 bit. Feature Extraction software 10.7 (Agilent technologies, Santa Clara, CA, US) Raw data were normalized by Quantile algorithm, Gene Spring Software 11.0 (Agilent technologies, Santa Clara, CA, US) (Table 1).

#### 2.7.5. Real-Time PCR

Primers of the four genes were designed with Primer Express 2.0 (Oebiotec, Shanghai, China) (Table 2). Reverse transcription was performed on PrimerScript RT reagent Kit (TaKaRa, DRR037A, Takara Biotechnology (Dalian) Co., Ltd. China). Total RNA (0.5 *μ*g) was denatured at room temperatrue then mixed with the reagent in a final volume of 10 *μ*L containing 50 *μ*M oligo dT, 100 *μ*M random primer, 0.5 mM dNTP and the manufacturer's buffer and Enzyme Mix. The RT reaction was conducted for 15 min at 37°C, and 85°C for 5 s in ABI 9700. First-strand cDNA product was diluted in 100 *μ*L distilled water in preparation for real-time PCR. qPCR was performed using SuperReal PreMix (SYBR Green) kit (TIANGEN, FP204, Tiangen Biotech (Beijing) Co., Ltd. Beijing, China). Briefly, 1 *μ*L of diluted cDNA product was used for 40-cycle three-step PCR in a Roche HOLD CYCLE LightCycler 480 II.

### 2.8. Statistical Analysis

The body development, behavioral test, and hormone level data were analyzed using a Statistical Package for the Social Sciences (SPSS) version 19.0. ANOVA for Repeated Measurement with Greenhouse-Geisser Adjustment was performed to analyze group differences in body weight. A nonparametric Mann-Whitney test was performed to analyze group differences on the OFT. Student's *t*-test was performed to analyze group differences in corticosterone, dopamine, 5-HT, and growth hormone. Alpha was set to.05 for all analyses.

## 3. Results

### 3.1. Body Development and Behavior Test

**Figure 1:**
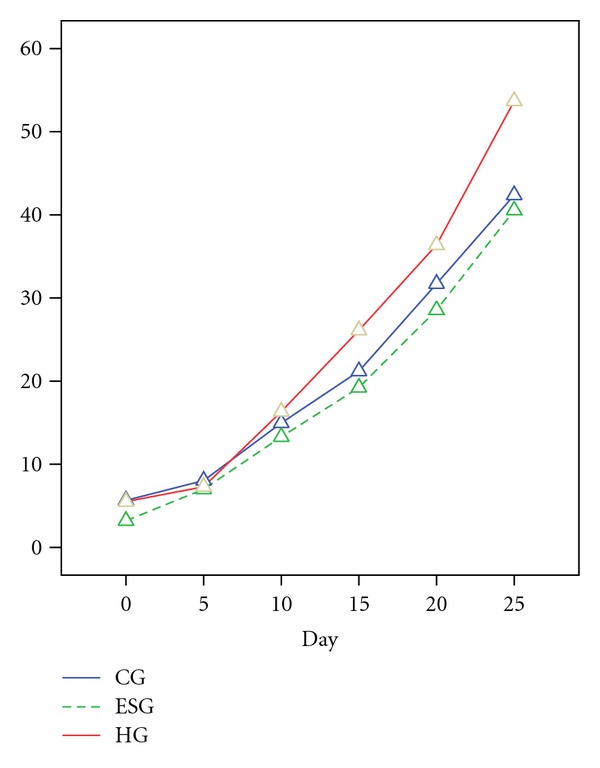
Mean plot of body weight. According to the ANOVA for Repeated Measurement, the body weight of ESG offspring were statistically all inferior to the CG offspring despite in Day 10 (*P* < 0.05). The body weight HG offspring were statistically superior to the ESG offspring despite in Day 5 (*P* < 0.05); The body weight HG in Day 15, Day 20 and Day 25 were statistically superior to the CG (*P* < 0.05).

ANOVA for Repeated Measurement with Greenhouse-Geisser Adjustment (Mauchly's *W* = 0.085, Approx. Chi-square = 214.490, df = 14, *P* ⩽ 0.001, Greenhouse-Geisser = 0.541) showed a statistically significant difference of the body weight of the 6 observation time spots of offspring among CG, ESG, and HG offspring (body weight: df = 2.705, mean square = 39791.256, *F* = 1923.553, *P* ⩽ 0.001; body weight∗group  df = 5.410, mean square = 415.400, *F* = 20.081, *P* ⩽ 0.001). Generally, HG offspring was heavier than CG, which is heavier than ESG (Figure 1).

**Figure 2:**
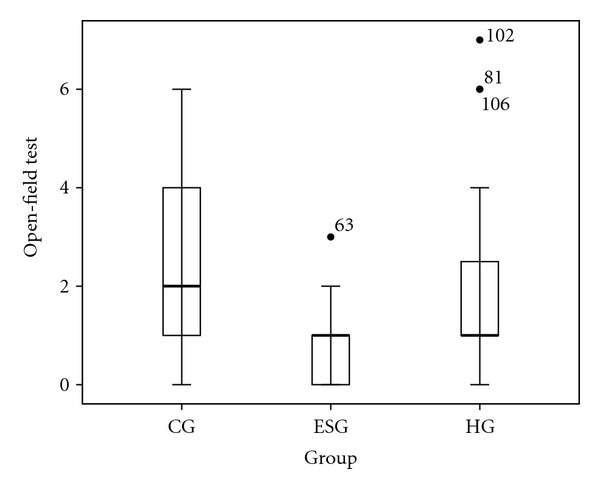
Box plot of OFT in the comparison between CG, ESG, and HG. ESG showed less scores than CG (*P* < 0.05) and HG (*P* < 0.05).

A Mann-Whitney test showed significant difference between the three groups on the OFT (Mann-Whitney *U* = 1448.500, Wilcoxon *W* = 2529.500, *Z* = −3.819, *P* = 0.000) (Figure 2): the OFT scores of HG and CG were both significantly higher than those observed in the ESG.

### 3.2. Hormone Levels

**Figure 3:**
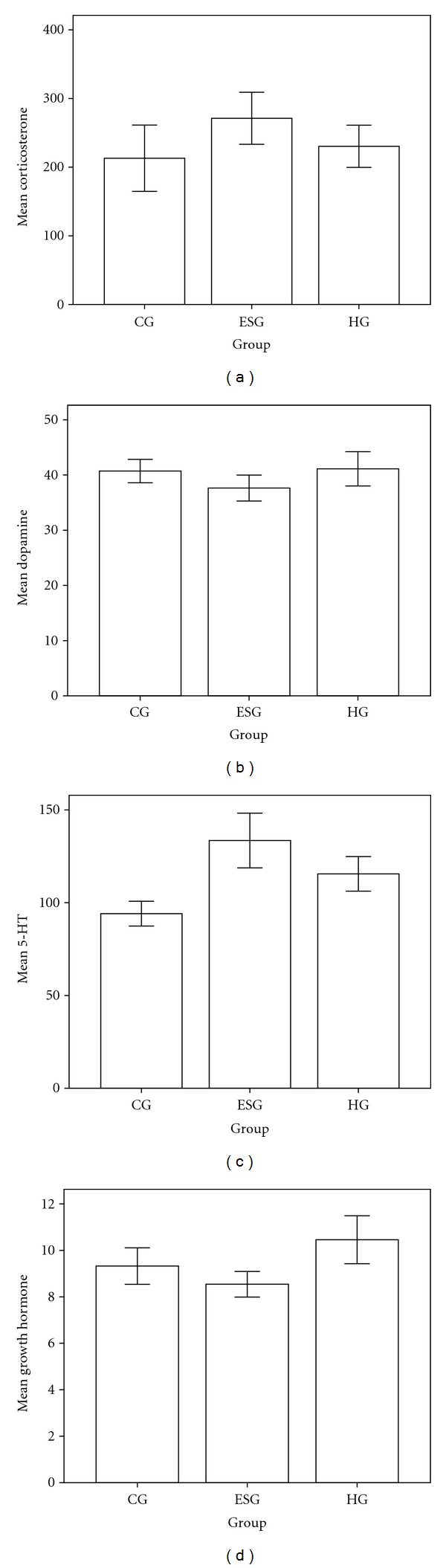
ELISA outcomes of corticosterone, dopamine, 5-HT, and growth hormone. (a) ANOVA test for the corticosterone showed *P* = 0.027 in CG versus ESG, *P* = 0.491 in CG versus HG, and *P* = 0.111 in ESG versus HG. (b) ANOVA test for the dopamine showed *P* = 0.065 in CG versus ESG, *P* = 0.805 in CG versus HG, and *P* = 0.039 in ESG versus HG. (c) ANOVA test for 5-HT showed *P* = 0.000 in CG versus ESG, *P* = 0.004 in CG versus HG, and *P* = 0.013 in ESG versus HG. (d) ANOVA test for the growth hormone showed *P* = 0.135 in CG versus ESG, *P* = 0.034 in CG versus HG, and *P* = 0.001 in ESG versus HG.

The corticosterone levels of CG was statistically lower than ESG and slightly than HG (Figure 3(a)). The dopamine level of ESG was slightly lower than the CG and of HG was significantly higher than the ESG (Figure 3(b)). The 5-HT of ESG showed a highest level and the CG lowest (Figure 3(c)). The growth hormone level of the HG was statistically higher than the CG and ESG (Figure 3(d)). 

### 3.3. Gene Expression Profile

#### 3.3.1. ESG versus CG

**Figure 4:**
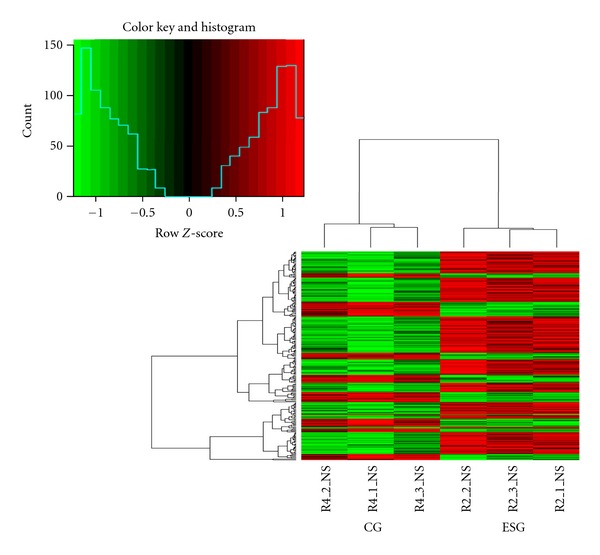
Heat map of the differently expressed genes. R2_1_NS, R2_2_NS, and R2_3_NS refer to ESG and R4_1_NS, R4_2_NS, R4_3_NS to CG.

Gene expression profile showed 81 genes upregulated and 39 genes downregulated (*P* < 0.01) in ESG versus CG comparison (Table 3 (see Supporting Information 1),Figure 4), among which 14 GO annotations were obtained including, ligase activity, regulation of metabolic process, positive regulation of metabolic process, cellular component assembly, membrane bounded organelle, biosynthetic process, cellular component biogenesis, and cellular response to stimulus. (Table 4 (Supporting Information 2)), and among which 12 KEGG pathways were annotated, including oocyte meiosis, vascular smooth muscle contraction, RIG-I-like receptor signaling pathway, long-term potentiation, ubiquitin mediated proteolysis, and long-term depression (Table 5).

#### 3.3.2. ESG versus HG

**Figure 5:**
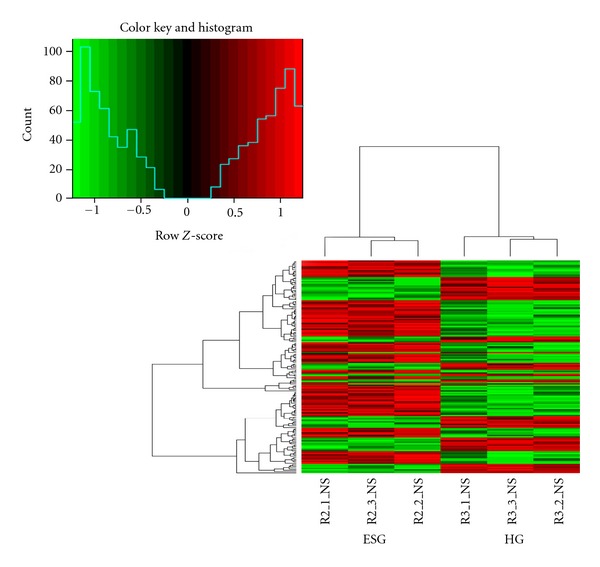
Heat map of the differently expressed genes. R2_1_NS, R2_2_NS, and R2_3_NS refer to ESG and R3_1_NS, R3_2_NS, R3_3_NS to HG.

Gene expression profile showed 60 genes upregulated and 28 genes downregulated (*P* < 0.01) in ESG versus CG (Table 6 (Supporting Information 3), Figure 5), among which five GO annotations were obtained including protein complex localization, cellular component assembly, cellular component biogenesis, anatomical structure formation, and organelle lumen (Table 7), and among which 5 KEGG pathways were annotated, including cell cycle, Jak-STAT signaling pathway, Type II diabetes mellitus, One carbon pool by folate, and insulin signaling pathway (Table 8).

No genes were found, which were significantly differently expressed simultaneously in ESG versus CG and ESG versus HG. However, 8,426 genes were found no statistical difference in HG versus CG (*P* > 0.05) among which 84 were found also presented in the differently expressed genes in ESG versus HG (Table 9 (Supporting Information 4)).

#### 3.3.3. RT-PCR Validation

**Figure 6:**
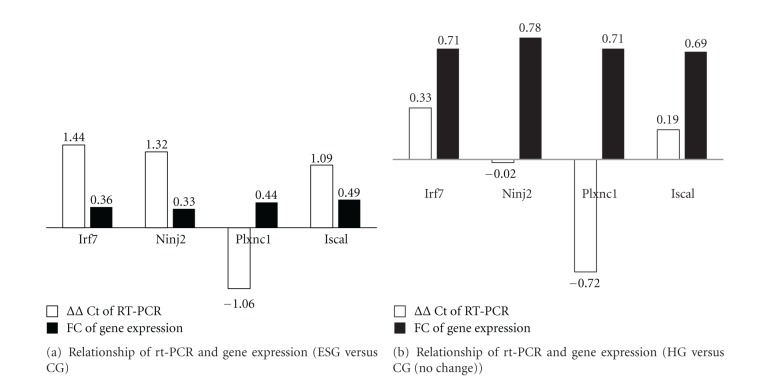
RT-PCR validation of the selected four genes from gene expression profile chips, that is, Irf7, Ninj2, and Plxnc1, and Isca1. ΔΔCt < 0 indicates the target genes were hyperexpressed in ESG/HG comparing with CG while ΔΔCt > 0 indicates the target genes were hypoexpressed in ESG/HG comparing with CG. FC > 2 indicates the target genes were hyperexpressed in ESG/HG comparing with CG while FC < 0.5 indicates the target genes were hypoexpressed in ESG/HG comparing with CG.

Irf7, Ninj2, Plxnc1, and Isca1 were filtered to validate with RT-PCR according to the set that the flag value of the expression profile chip *≠A*, FC > 2 or FC < 0.5, expression value ≥6 from the GO and KEGG annotation. As showed in Figure 6(a), Irf7, Ninj2, and Isca1 were significantly hypoexpressed in ESG (FC < 0.5); however, the gene expression of Plxnc1 did not match the RT-PCR validation; in Figure 6(b), the four genes were not significantly hypoexpressed in HG versus CG (0.5 < FC < 2), and the RT-PCR validation showed an obviously reduced ΔΔCt values compared with those in Figure 6(a). The gene expression profile chip outcomes showed a favorable match with the RT-PCR result.

## 4. Discussion

Substantial evidence from preclinical laboratory studies indicates that PS affects the hormonal and behavioral development of offspring. PS has been found to alter baseline and stress-induced responsivity of the HPA axis and levels and distribution of regulatory neurotransmitters, such as norepinepherine, dopamine, serotonin, and acetylcholine and to modify key limbic structures and to retard intrauterine growth [15]. In this study, ESG demonstrated differences from CG on body weight, hormone levels, and gene expressions, and HG differed from the ESG group on body weight, hormone levels, and gene expressions. From the perspective of Chinese medicine, once parental kidney is injured from PS, manifestations are handed down to offspring, showing development retardation and OFT performance reduction. JKSQW is a typical herbal formula for kidney qi supplementing, which recovers the physiological functions of kidney. In this study, the body weight and OFT performance were improved by JKSQW, supporting the effectiveness of Chinese herb remedy in rodents in lab [13].

Experimentally, PS in animal models mal-programs offspring physiology, resulting in increasing the likelihood of disorders of HPA axis activity and anxiety-related behaviors in adulthood [16]. PS increases plasma levels of corticosterone and corticotrophin releasing hormone in the mother and fetus, which may contribute to insulin resistance and behavior disorders in their offspring that include attention and learning deficits, generalized anxiety and depression [17]. We demonstrated that the serum corticosterone of ESG were significantly higher than CG and slightly higher than HG, which was in accordance with previous reports [18–20]. Animal studies indicate that PS can affect the activity of the placental barrier enzyme 11-*β*HSD2 (11*β*-hydroxysteroid dehydrogenase type 2), which metabolizes corticosterone [2, 17]. 5-HT level of ESG was significantly higher than CG and HG. Alterations in activity of the central 5-HT system play an essential role in many of these behavioral aberrations due to PS [21, 22]. During pregnancy, the 5-HT system has a fundamental role in the fetus' development of the central nervous system, and 5-HT neurotransmission is involved in the activation and feedback of HPA axis throughout life [23]. Huang et al. [14] reported that levels of 5-HT were higher in rat hippocampus and hypothalamus of fetuses in the CUS group, that is, chronic unpredictable stress maternally performed than in the controls. Increased 5-HT signaling increases the expression of key transcription factors, notably nerve growth factor induced protein A, which binds to and regulates activation of the GR promoter [24]. No difference of the dopamine level between ESG and CG were obtained, indicating earthquake may not alter the offspring dopamine. Interestingly JKSQW in HG significantly elevated the dopamine level of ESG. Carboni et al. [25] reported prenatal catecholamine stimulation was obtained by amphetamine or nicotine. We observed that PS did not change dopamine. No difference of the hormone level between ESG and CG were obtained, indicating earthquake may not impact on the growth hormone of offspring. Interestingly, however, JKSQW in HG significantly elevated the dopamine level of ESG, which might be explained by the function of kidney that governs development. Shen and Cai [26] reported that growth hormone genes were downregulated in a kidney-qi deficiency rat model and Chinese formula supplementing kidney qi could correct the downregulation. Mak et al. [27] found that chronic kidney disease in children was associated with dramatic changes in the growth hormone and insulin-like growth factor (IGF-1) axis, resulting in growth retardation. Yang and Li [28] reported that JKSQW could recover the downregulated growth hormone genes (Somatotropin precursor, NM-008117) in a kidney-yang deficiency rat model. Researches of the neurobiological mechanisms underlying the interaction between PS and adult mental disorders suggest the involvement of multiple neurotransmitter systems [29, 30]. Findings of the hormones alterations suggest manual earthquake is a liable model modulating the fear from natural earthquake involving development retardation and neurotransmitter systems disorder. Meanwhile, from the perspective of Chinese medicine, kidney function is disturbed by the earthquake and recovered by JKSQW.

We found 81 genes upregulated and 39 genes downregulated in ESG versus CG, from which 14 significant GO and 12 KEGG pathways were annotated, indicating diversified and complicated physiological and psychological impacts on offspring left by the prenatal earthquake as a prenatal stress, for example, long-term depression and long-term potentiation. Mychasiuk et al. [31] reported that significant gene expression level changes in 558 different genes, associated with overrepresentation of 36 biological processes and 34 canonical pathways indicating prenatal stress did not have to be experienced by the mother herself to influence offspring brain development. Among the GO annotations Itpr1 and Itpr2 appeared in almost all the affected pathways. In nonexcitable cells, the inositol 1,4,5-trisphosphate receptor (IP3R) is an intracellular Ca^2C^ channel, which plays a major role in Ca^2C^ signalling. Three isoforms of IP3R have been identified (IP3R-1, IP3R-2, and IP3R-3) and most cell types express different proportions of each isoform [31]. IP3Rs play major roles in agonists-induced intracellular Ca^2C^ release and also in store operated Ca^2C^ entry, a process whereby the depletion of intracellular Ca^2C^ store causes the opening of Ca^2C^ channels in the plasma membrane [32]. The intracellular Ca^2+^ elevations induced by BDNF required a signaling pathway consistent with the activation of the Trk-IP3R cascade, which was also necessary for the activation of the membrane conductance IBDNF [33, 34]. Amaral and Pozzo-Miller [35] reported that Trk receptors, IP3Rs, full intracellular Ca^2+^ stores and Ca^2+^ influx are all required for BDNF-induced Ca^2+^ elevations and membrane currents. Opposing influences of mBDNF and proBDNF on long-term potentiation and long-term depression might contribute to the dichotomy of BDNF actions on behaviors mediated by the brain stress and reward systems [36, 37]. Twelve KEGG pathways were annotated, including oocyte meiosis, vascular smooth muscle contraction, RIG-I-like receptor signaling pathway, long-term potentiation, ubiquitin mediated proteolysis, and long-term depression, Titterness and Christie [38] prenatal ethanol and prenatal stress produce sex-specific alterations in synaptic plasticity in the adolescent hippocampus. Calpains, which belong to a family of at least 14 members of calcium-dependent cysteine proteases and are involved in apoptosis are implicated in a wide range of physiological functions including cell motility, differentiation, signal transduction, including cell survival pathways, cell cycle progression, regulation of gene expression, and long-term potentiation [39, 40]. Yang et al. [41] reported that prenatal stress (10 unpredictable, 1 s, 0.8 mA foot shocks per day during gestational days 13–19) impaired long-term potentiation (LTP) but facilitated long-term depression (LTD) in hippocampal CA1 region in slices of the prenatal stressed offspring (5 weeks old). Proteolysis by the ubiquitin-proteasome pathway has attained prominence as a new molecular mechanism which regulates varied important functions of the nervous system, including development of synaptic connections and synaptic plasticity through control of axonal growth, axonal and dendritic pruning, and regulation of synaptic size and number [42]. 

We found 60 genes upregulated and 28 genes downregulated in HG versus ESG, from which five significant GO and five KEGG pathways were annotated, indicating diversified cellular biological process and signaling pathways. Interestingly, Socs 2 and Socs 4 of Socs (suppressors of cytokine signaling) family appeared in three of the KEGG pathways. SOCS family consists of eight structurally similar proteins (SOCS-1 to SOCS-7 and CIS), which have been implicated as potential inhibitors of tissue growth during both prenatal and postnatal life [43] and their actions clearly now extend to other intracellular pathways, they remain key negative regulators of cytokine and growth factor signaling [44]. Cytokine-mediated JAK/STAT signaling, that is, Janus kinase/signal transducers and activators of transcription, controls a number of vital biologic responses, including immune function, cellular growth, differentiation, and hematopoiesis [45]. The SOCS Family—The SOCS proteins were identified as STAT target genes that directly antagonize STAT activation, resulting in a classic “feedback loop” [46]. PS in rats induced lifespan reduction of neurogenesis in the dentate gyrus and produced impairment in hippocampal-related spatial tasks through blocking the increase of learning-induced neurogenesis [47]. Previous research reported that male rats exposed to stress in utero are characterized by a decrease in hippocampal cell proliferation, and consequently neurogenesis, from adolescence to senescence [48]. PS has been reported to alter cytokine levels. Coussons-Read et al. [49] reported that stress-related neural immune interactions may contribute to pregnancy complications and poor outcome. Collier et al. [50] found that PS changed typical proinflammatory cytokines including tumor necrosis factor (TNF)-*α*, and interleukin (IL)-6. As mentioned above, JKSQW recovered the dysfunction of kidney due to fear from earthquake, which could be supported by gene profile experiment outcome. In other words, cytokine conduction pathways, for example, JAK/STAT are involved in the prenatal kidney deficiency, and key molecules like Socs-2 and Socs-4 are the regulating targets of Chinese medicine treatment. The underlying mechanism that JKSQW improves development and behavior might attribute to the upregulation of Socs-2 and Socs-4 which suppress the pathway of JAK/STAT, resulting in reduction certain cytokines' secretion. diabetes is considered as Xiao-ke in Chinese medicine, whose major pattern is kidney deficiency. JKSQW plays an important role in the composition of prescriptions treating Diabetes in Chinese medicine [51]. Promisingly, our findings revealed insulin related pathways were involved in the outcome of herbal intervention in HG, supporting the hypnosis that JKSQW recovery the dysfunction of kidney.

Four genes (Irf7, Ninj2, Plxnc1, and Isca1) were validated with RT-PCR, showing a favorable match (75%) between the gene expression profile chip and RT-PCR result. It is reported that all elements of IFN responses, whether the systemic production of IFN in innate immunity or the local action of IFN from plasmacytoid dendritic cells in adaptive immunity, are under the control of Irf7 [52]. Hannah et al. [53] reported that induction of pattern recognition receptors (PRRs; Tlr7 and Rig-I), expression of antiviral genes (Myd88, Visa, Jun, Irf7, Ifnbeta, Ifnar1, Jak2, Stat3, and Mx2), and production of Mx protein was elevated in the lungs of intact females compared with intact males. Ninjurin2 (Ninj2) is a transmembrane protein that mediates cell-to-cell and cell-to-extracellular matrix interactions during development, differentiation, and regeneration of the nervous system [54]. Recently, Ninj2 was reported to be a vascular susceptibility gene and associated with Alzheimer's disease risk [55]. 

In conclusion, together with our own recent data, the findings of this body of work demonstrate the earthquake as a prenatal stressor during the pregnancy could negatively retard the body and nervous system development, and Chinese herbal remedy could correct the retardation, which could attribute to neurohormones alteration and altered gene expression profile. The gene pathways involved have been tied to signaling pathway, long-term potentiation, ubiquitin mediated proteolysis, and long-term depression relating to disruptions from prenatal stress; Jak-STAT signaling pathway could play a key role in improving the function of JKSQW. This study demonstrates that negatively prenatal experiences have the ability to significantly retard offspring developmental and immunity trajectories, which can be corrected by Chinese herbal remedy.

## Figures and Tables

**Table 1:** QC of RNA extraction and slides experiment (A sample is qualified only when 2100 RIN ≥ 7.0 and 28S/18S ≥ 0.7).

Group	QC of RNA							QC of slides	
	Con. (*μ*g/*μ*L)	Vol. (*μ*L)	Total (*μ*g)	A260/A280	2100 Result		Result	CV (%)*	Detection rate (%)
					RIN	28S/18S			
ESG	0.168	50	8.41	1.88	9.4	1.7	Qualified	3.91	69.50
	1.366	30	40.98	1.93	9.5	1.8	Qualified	4.85	62.96
	0.246	50	12.29	1.90	9.4	1.7	Qualified	6.70	72.13
HG	0.134	50	6.69	1.81	9.4	1.8	Qualified	4.76	70.40
	0.138	50	6.92	1.82	9.4	1.8	Qualified	4.90	72.30
	0.372	50	18.58	1.86	9.5	1.6	Qualified	4.89	69.43
CG	0.185	50	9.27	1.91	9.4	1.7	Qualified	6.33	61.09
	0.595	50	29.75	1.93	9.4	1.7	Qualified	5.70	70.68
	0.355	25	8.87	1.85	9.3	1.6	Qualified	4.39	65.52

*CV = SD/Mean × 100%.

**Table 2:** Primers and product length of the four targeted genes.

No.	Gene symbol	Forward primer	Reverse primer	Product length
1	*ACTB	GCGTCCACCCGCGAGTACAA	ACATGCCGGAGCCGTTGTCG	118
2	Irf7	TGGCAGATGGAAGCTACC	GGCTATACAGGAACACGC	154
3	Ninj2	CCACCACCTTGGTCTTCATA	AGGCTGAAGTGGCTTTAG	152
4	Isca1	CCCGTTGCATCTTTACCAC	GTCTAAGCAAACCGCATGAA	151
5	Plxnc1	TGACCACTGCCACTTGAT	CTGAAGAGTTTCTCAAGCAC	159

*refers to internal control gene.

**Table 3:** Differentially expressed genes in ESG versus CG, among which 39 genes were upregulated and 81 genes downregulated.

Gene ID	*P* values	Fold change	Gene symbol	Regulation
63847	0.007006	0.096204	Fxyd6	Downregulated
498145	0.003225	0.17368	LOC498145	Downregulated
316628	0.004414	0.274831	Asb1	Downregulated
360547	0.005836	0.320844	Sat2	Downregulated
301245	0.007067	0.331729	Yipf3	Downregulated
293023	0.009502	0.335662	Klhl25	Downregulated
288240	0.002174	0.344925	Hlcs	Downregulated
293180	0.007695	0.352823	Micalcl	Downregulated
316426	0.003961	0.363248	Spats2l	Downregulated
293624	0.008043	0.364195	Irf7	Downregulated
683788	0.007907	0.382175	LOC683788	Downregulated
293156	0.009012	0.413953	Lrtomt	Downregulated
25646	0.004102	0.429726	Otx1	Downregulated
290232	0.009311	0.430944	Tinf2	Downregulated
498353	0.002896	0.440115	Scfd2	Downregulated
362873	0.006203	0.440433	Plxnc1	Downregulated
309415	0.009479	0.458925	Fam189a2	Downregulated
113894	0.007725	0.463149	Sqstm1	Downregulated
303538	0.003261	0.465171	Dhx58	Downregulated
406196	0.001118	0.467157	Hcr	Downregulated
313917	0.005676	0.482298	Abhd1	Downregulated
292811	0.009904	0.48439	Ccdc123	Downregulated
290985	0.007918	0.491881	Isca1	Downregulated
405152	0.008771	0.516648	Olr1192	Downregulated
171355	0.005274	0.519609	Pou4f2	Downregulated
362943	0.000172	0.526926	Adck5	Downregulated
309161	0.001612	0.543788	Ccdc85b	Downregulated
361327	0.003693	0.596748	Prr16	Downregulated
24640	0.008865	0.602226	Pfkfb2	Downregulated
619573	0.006811	0.603084	Fam104a	Downregulated
116725	0.007447	0.653258	Ube2n	Downregulated
304342	0.005141	0.662423	Zscan21	Downregulated
192252	0.009069	0.671766	Dctpp1	Downregulated
114205	0.00295	0.677239	Crcp	Downregulated
311430	0.007769	0.689602	Mavs	Downregulated
287840	0.003671	0.716317	Fam100b	Downregulated
297109	0.006823	0.764608	MGC95152	Downregulated
295037	0.000491	0.788096	Mgst2	Downregulated
100360990	0.007759	0.815928	LOC100360990	Downregulated
501083	0.00564	1.179002	Pdcd6ip	Upregulated
299195	0.000513	1.189394	Coq6	Upregulated
81716	0.007768	1.20684	Ggcx	Upregulated
315023	0.008157	1.265746	Slc25a32	Upregulated
296753	0.009238	1.284846	Srpk2	Upregulated
299147	0.005455	1.304917	Ppp2r5e	Upregulated
361932	0.009554	1.307515	RGD1561393	Upregulated
288259	0.009614	1.31293	Gart	Upregulated
289522	0.002341	1.325268	Cox18	Upregulated
50688	0.002132	1.334825	Cacnb1	Upregulated
363171	0.000593	1.337206	Tmem42	Upregulated
114215	0.005997	1.352079	Insl3	Upregulated
315771	0.008317	1.369011	Herc1	Upregulated
360389	0.009442	1.375028	Zfp422	Upregulated
305923	0.008185	1.393988	Zdhhc20	Upregulated
24803	0.005163	1.399617	Vamp2	Upregulated
363210	0.001697	1.411325	Phf3	Upregulated
50561	0.001722	1.425023	Resp18	Upregulated
362367	0.005441	1.43527	Znrf2	Upregulated
170841	0.009557	1.458549	Mutyh	Upregulated
81678	0.003588	1.464706	Itpr2	Upregulated
502886	0.009395	1.466283	Foxj2	Upregulated
360868	0.009274	1.471063	Sft2d2	Upregulated
313757	0.005281	1.485264	RGD1565591	Upregulated
361109	0.000669	1.486251	Dcp1a	Upregulated
192210	0.008713	1.487999	Dnajc21	Upregulated
25262	0.008127	1.49478	Itpr1	Upregulated
311112	0.00906	1.533447	Fastkd1	Upregulated
64086	0.004012	1.55121	Csnk1g1	Upregulated
366693	0.007515	1.567923	Rbm25	Upregulated
690961	0.006894	1.577038	Cog2	Upregulated
292148	0.004257	1.589999	Eif3a	Upregulated
691918	0.002531	1.596744	LOC691918	Upregulated
362317	0.001503	1.599092	Krit1	Upregulated
54323	0.001154	1.610286	Arc	Upregulated
304813	0.005676	1.614358	Ppp1r12b	Upregulated
58983	0.00216	1.617294	Rabggta	Upregulated
361944	0.004739	1.617335	Elf2	Upregulated
314862	0.000215	1.618023	Dyrk2	Upregulated
29642	0.003006	1.62079	Slc38a2	Upregulated
291409	0.00357	1.622726	Zfp236	Upregulated
246282	0.001061	1.623318	Zfp91	Upregulated
362132	0.00226	1.626565	Epc2	Upregulated
303963	0.002236	1.631518	Dzip3	Upregulated
116670	0.006773	1.634179	Ppp1r12a	Upregulated
302670	0.004529	1.63737	Zrsr2	Upregulated
360993	0.006601	1.637448	Smek2	Upregulated
59319	0.001208	1.6438	Nyw1	Upregulated
287249	0.009286	1.659325	Cnot6	Upregulated
362132	0.007917	1.663529	Epc2	Upregulated
303511	0.004368	1.665157	Ikzf3	Upregulated
363210	0.008478	1.665263	Phf3	Upregulated
362096	0.00268	1.668933	Setx	Upregulated
316583	0.001117	1.700923	B3gnt7	Upregulated
362817	0.008175	1.701909	Cdk2	Upregulated
304157	0.009185	1.708222	Nrip1	Upregulated
314169	0.009008	1.729076	Fam179b	Upregulated
303919	0.007784	1.731828	Lrrc58	Upregulated
309523	0.005447	1.734164	Kif20b	Upregulated
291773	0.003136	1.741424	RGD1562997	Upregulated
314423	0.003545	1.743689	Bcl11b	Upregulated
362622	0.007916	1.756522	Ccdc21	Upregulated
497198	0.005781	1.770803	Impact	Upregulated
315804	0.00029	1.773739	Rfx7	Upregulated
363287	0.002339	1.775948	Hdac4	Upregulated
361688	0.00606	1.778637	Suv420h1	Upregulated
363555	0.002239	1.787221	Wfikkn1	Upregulated
304809	0.001337	1.791911	Kdm5b	Upregulated
498803	0.003675	1.797804	Otud1	Upregulated
64624	0.005484	1.803225	Cul5	Upregulated
304817	0.00381	1.807047	Ipo9	Upregulated
54311	0.008729	1.82334	Timm17a	Upregulated
25486	0.008651	1.8782	Myo9b	Upregulated
302612	0.006615	1.978189	Tspyl2	Upregulated
293765	0.003013	2.076238	Olr327	Upregulated
171347	0.007854	2.324322	Mat2a	Upregulated
685074	0.008629	2.417108	LOC685074	Upregulated
498211	0.007458	2.449546	RGD1560523	Upregulated
690043	0.004624	2.470614	Rnf168	Upregulated
171347	0.00179	2.47901	Mat2a	Upregulated
363083	0.007379	2.521284	Fbxl22	Upregulated

**Table 4:** Significant GO annotation of the 120 differentially expressed genes and the genes involved (*P* < 0.05).

GO Id	Name	Symbol	Hits	Total	Percent	Enrichment test *P* value
GO: 0016874	Ligase activity	Ube2n, Hlcs				
		Gart, Herc1,	7	308	2.27%	0.0083
		Cul5, Rnf168, Ggcx				

GO: 0019222	Regulation of metabolic process	Sqstm1, Insl3, Ube2n, Pou4f2,				
		Otx1, Cnot6, Tinf2,				
		RGD1562997, Irf7,				
		Tspyl2, Nrip1,	28	2415	1.16%	0.0089
		Zscan21, Jarid1b, Bcl11b, Dyrk2,				
		Mll1, Rfx7, Zfp422, Smek2, Suv420h1, Elf2, Cdk2, Hdac4, Impact,				
		Foxj2, Rasd1, Rnf168, Pfn2				

GO: 0009893	Positive regulation of metabolic process	Sqstm1, Insl3, Ube2n,				
		Pou4f2, Tinf2,				
		Nrip1, Zscan21, Bcl11b	13	846	1.54%	0.0098
		Dyrk2, Mll1, Cdk2				
		Hdac4, Rnf168				

GO: 0022607	Cellular component assembly	Sqstm1, Xtp3tpa				
		Vamp2, Cox18, Tinf2, Eif3s10, RGD1562997	12	786	1.53%	0.0135
		Srpk2, Mll1, Enth, Pfn2				

GO: 0043227	Membrane-bounded organelle	Sqstm1, Crcp, Ube2n, Mutyh, Pou4f2, Vamp2				
		Itpr1, Otx1, Cnot6				
		Hlcs, Cox18, Tinf2				
		Isca1, Eif3s10				
		RGD1562997				
		Irf7, Srpk2, Ikzf3				
		Ppp2r5e, Yipf3				
		Tspyl2, Zrsr2, Nrip1				
		Zscan21, Kif20b				
		Visa, RGD1565591	55	5982	0.92%	0.025
		Bcl11b, Dyrk2				
		Slc25a32, Mll1, Enth				
		B3gnt7, Zfp422, Setx				
		Suv420h1, Elf2, Phf3				
		Cdk2, Adck5, Hdac4 Hcr, LOC498145				
		Pdcd6ip, Foxj2, Rasd1, Resp18, Cul5				
		Cacnb1,Timm17a,				
		Arc, Rnf168, Cog2,			
		Itpr2, Ggcx			

GO: 0014854	Response to inactivity	Hdac4	1	3	33.33%	0.0288

GO: 0009058	Biosynthetic process	Crcp, Insl3,Ube2n				
		Mat2a, Pou4f2, Otx1				
		Cnot6, Gart, Tinf2				
		Isca1, RGD1562997				
		Eif3s10, Irf7, Coq6				
		Tspyl2, Nrip1, Mll1	34	3379	1.01%	0.0291
		Zscan21, Jarid1b				
		Bcl11b, Dyrk2, Rfx7				
		B3gnt7, Zfp422, Elf2				
		Suv420h1, Cdk2, Phf3				
		Hdac4, Impact, Foxj2				
		Rabggta, Rasd1				

GO: 0044085	Cellular component biogenesis	Sqstm1, Xtp3tpa,				
		Vamp2, Cox18, Tinf2				
		RGD1562997, Eif3s10	12	883	1.36%	0.0299
		Srpk2, Mll1, Enth, Pfn2				

GO: 0014874	Response to stimulus involved in regulation of muscle adaptation	Hdac4	1	4	25.00%	0.0359

GO: 0043233	Organelle lumen	Sqstm1, Mutyh, Itpr1				
		Tinf2, RGD1562997				
		Srpk2, Tspyl2, Zrsr2	16	1360	1.18%	0.0416
		Nrip1, Kif20b, Mll1				
		Zfp422, Setx, Cdk2				
		Hdac4, Resp18				

GO: 0051716	Cellular response to stimulus	Ube2n, Mutyh, Dyrk2				
		Mll1, Setx, Cdk2,	8	528	1.52%	0.0422
		Pdcd6ip, Rnf168				

GO: 0016740	Transferase activity	Crcp, Mat2a, Pfkfb2				
		Gart, Mgst2, Srpk2				
		RGD1304822, Dyrk2	18	1612	1.12%	0.0483
		Fastkd1, Mll1, B3gnt7				
		Suv420h1, Cdk2, Fgfr1l, RGD1560523				
		Rabggta, Csnk1g1				

GO: 0031974	Membrane enclosed lumen	Sqstm1, Mutyh, Itpr1				
		Tinf2, RGD1562997				
		Srpk2, Tspyl2, Zrsr2				
		Nrip1, Kif20b, Mll1	16	1392	1.15%	0.0495
		Zfp422, Setx, Cdk2,				
		Hdac4, Resp18				

GO: 0031077	Postembryonic camera-type eye development	Bcl11b	1	6	16.67%	0.0499

**Table 5:** KEGG Pathway annotation of the 120 differentially expressed genes (*P* < 0.05, *q* < 0.05) (↓ refers downregulation, ↑ refers upregulation).

Name	Symbol	Total	Percent	Enrichment test *P* value	*q* value
Oocyte meiosis	Itpr1↑ Ppp2r5e↑	116	0.0345	0.0008	0.0048
	Cdk2↑				
Vascular smooth muscle contraction	Ppp1r12a↑	128	0.0313	0.0011	0.0048
	Itpr1↑				
	Ppp1r12b↑				
RIG-I-like receptor signaling pathway	Irf7↓ Dhx58↓	64	0.0469	0.0016	0.0048
	Mavs↓				
Long-term potentiation	Ppp1r12a↑ Itpr1↑	72	0.0417	0.0022	0.0049
	Itpr2↑				
Ubiquitin mediated proteolysis	Ube2n↓ Herc1↑	132	0.0227	0.0111	0.0176
	Cul5↑				
Cytosolic DNA-sensing pathway	Irf7↓ Mavs↓	49	0.0408	0.0131	0.0176
Biotin metabolism	Hlcs↓	3	0.3333	0.0135	0.0176
RNA degradation	Cnot6↑ Dcp1a↑	61	0.0328	0.0196	0.0223
Long-term depression	Itpr1↑ Itpr2↑	69	0.029	0.0245	0.0245
Ubiquinone and other terpenoid-quinone biosynthesis	Coq6↑	7	0.1429	0.0269	0.0245
Phosphatidylinositol signaling system	Itpr2↑ Itpr1↑	77	0.026	0.0299	0.0247
Gap junction	Itpr2↑ Itpr1↑	87	0.023	0.0371	0.0281
GnRH signaling pathway	Itpr1↑ Itpr2↑	99	0.0202	0.0467	0.0326

**Table 6:** Differentially expressed genes in ESG versus HG, among which 60 genes were upregulated and 28 genes downregulated.

Gene ID	*P* values	Fold change	Symbol	Remark
287881	0.006042	0.220799	Dysfip1	Downregulated
25405	0.004824	0.344631	Ccng1	Downregulated
24237	0.003207	0.40894	C6	Downregulated
313219	0.003811	0.410283	Zfp189	Downregulated
287343	0.008194	0.499299	Olr1454	Downregulated
293156	0.008272	0.508908	Lrtomt	Downregulated
405143	0.009972	0.5345	Olr803	Downregulated
116724	0.000512	0.546672	Epb4.1l3	Downregulated
313917	0.00383	0.578297	Abhd1	Downregulated
83681	0.004251	0.581219	Cish	Downregulated
301346	0.007628	0.609505	Sema4c	Downregulated
315346	0.003519	0.619843	Itga5	Downregulated
56825	0.009009	0.625224	Cym	Downregulated
690810	0.007066	0.637375	Adat1	Downregulated
313982	0.009162	0.653927	RGD1561890	Downregulated
363285	0.004745	0.660307	Scly	Downregulated
316090	0.003533	0.683347	Fam198a	Downregulated
24513	0.003494	0.687818	Ivd	Downregulated
303384	0.007792	0.703077	Mmp28	Downregulated
246074	0.009445	0.718762	Scd1	Downregulated
500011	0.008188	0.726294	RGD1563091	Downregulated
362943	0.004839	0.735253	Adck5	Downregulated
500420	0.008119	0.744282	LOC500420	Downregulated
399489	0.006413	0.763541	E2f1	Downregulated
311716	0.004912	0.77549	Col20a1	Downregulated
113894	0.007846	0.78406	Sqstm1	Downregulated
266609	0.005228	0.798742	Bles03	Downregulated
246766	0.00514	0.821038	Ggta1	Downregulated
288518	0.008613	1.136098	RGD1311660	Upregulated
499430	0.008063	1.148146	Lrrc20	Upregulated
317399	0.000156	1.156541	Ddx21	Upregulated
306182	0.00808	1.160148	Ipo5	Upregulated
301038	0.00729	1.178184	Ubp1	Upregulated
310806	0.006399	1.178549	Cdc14a	Upregulated
287954	0.003091	1.181263	Dgcr8	Upregulated
260321	0.008611	1.181875	Fkbp4	Upregulated
305828	0.006609	1.182203	Socs4	Upregulated
64161	0.005932	1.183779	Pi4ka	Upregulated
290679	0.009165	1.186593	Ints10	Upregulated
298429	0.006198	1.188777	Rad54l	Upregulated
474154	0.005077	1.190852	Rbm4b	Upregulated
288717	0.006268	1.196619	Srrd	Upregulated
296312	0.004568	1.197256	RGD1311066	Upregulated
312640	0.005739	1.198178	Tmem111	Upregulated
83624	0.009311	1.200882	Ppig	Upregulated
288778	0.001749	1.22319	Pa2g4	Upregulated
362851	0.004166	1.224723	Cd320	Upregulated
308404	0.006579	1.227818	Irf2bp1	Upregulated
363760	0.005704	1.237527	Arl6	Upregulated
296076	0.007529	1.238081	Srp14	Upregulated
291787	6.57*E*–05	1.242186	Rbbp8	Upregulated
500727	0.00344	1.246021	Cdca4	Upregulated
306587	0.008906	1.255527	Tcta	Upregulated
29541	0.000917	1.259108	Nthl1	Upregulated
360855	0.004605	1.26267	Smg7	Upregulated
362317	0.008649	1.284527	Krit1	Upregulated
313757	0.004801	1.294664	RGD1565591	Upregulated
499370	0.009663	1.326682	Itprip	Upregulated
288259	0.009472	1.335197	Gart	Upregulated
29704	0.002213	1.349013	Pacsin1	Upregulated
84472	0.006393	1.366251	Ilf3	Upregulated
363210	0.006023	1.388566	Phf3	Upregulated
680451	0.005563	1.419061	Nrbp2	Upregulated
311112	0.001699	1.426768	Fastkd1	Upregulated
54323	0.001608	1.4509	Arc	Upregulated
309136	0.006405	1.452428	Oraov1	Upregulated
363169	0.005748	1.472567	Toag1	Upregulated
29642	0.004937	1.475875	Slc38a2	Upregulated
305461	0.004104	1.475879	Fam53a	Upregulated
304813	0.00934	1.481691	Ppp1r12b	Upregulated
680006	0.007932	1.484512	Mad1l1	Upregulated
304474	0.001635	1.497221	Pitpnm2	Upregulated
115768	0.009088	1.509009	Zfp37	Upregulated
301513	0.001268	1.512431	Rqcd1	Upregulated
363273	0.009331	1.521116	Cops7b	Upregulated
293511	0.008749	1.533752	Znf688	Upregulated
245966	0.004372	1.544613	Tmem150a	Upregulated
291409	0.003844	1.552189	Zfp236	Upregulated
84607	0.007931	1.552588	Socs2	Upregulated
306344	0.007778	1.569477	Arrdc2	Upregulated
309828	0.006302	1.584851	Tspyl4	Upregulated
501095	0.009284	1.589281	Rftn1	Upregulated
81531	0.008017	1.606129	Pfn2	Upregulated
293152	0.007896	1.613085	Art2b	Upregulated
497040	0.006162	1.71037	Prss36	Upregulated
171454	0.009816	1.850404	Nacc1	Upregulated
363827	0.00216	1.948295	LOC363827	Upregulated
364361	0.001905	4.479744	RGD1563700	Upregulated

**Table 7:** Significant GO Annotation of the 5 differentially expressed genes and the genes included (*P* < 0.05).

GO ID	Name	Symbol	Hits	Total	Percent	Enrichment test *P* value
GO: 0031503	Protein complex localization	Fkbp4	1	5	20.00%	0.0309
GO: 0022607	Cellular component assembly	Sqstm1, Nacc1, Ivd, Fkbp4, Tspyl4, Itga5, Pfn2	8	786	1.02%	0.0548
GO: 0044085	Cellular component biogenesis	Sqstm1, Nacc1, Ivd, Fkbp4, Tspyl4, Itga5, Pfn2	8	883	0.91%	0.0926
GO: 0010926	Anatomical structure formation	Sqstm1, Nacc1, Ivd, Fkbp4, Ubp1, Tspyl4, Itga5, Pfn2	9	1049	0.86%	0.0993
GO: 0043233	Organelle lumen	Sqstm1, Nacc1, Ivd, Fkbp4, Pa2g4, Ints10, Nthl1, Ddx21, E2f1, Rbm4b, Ppig	11	1360	0.81%	0.0994

**Table 8:** KEGG Pathway annotation of the 120 differentially expressed genes (*P* < 0.05,  *q* < 0.05) (↓ refers downregulation, ↑ refers upregulation).

Name	Symbol	Total	Percent	Enrichment test *P* value	*q* value
Cell cycle	Cdc14a↑	132	0.0227	0.0044	0.0067
	E2f1↓				
	Mad1l1↑				
Jak-STAT signaling pathway	Socs4↑	149	0.0201	0.0062	0.0067
	Cish↓				
	Socs2↑				
Type II diabetes mellitus	Socs4↑	53	0.0377	0.008	0.0067
	Socs2↑				
One carbon pool by folate	Gart↑	17	0.0588	0.0429	0.0158
Insulin signaling pathway	Socs4↑	140	0.0143	0.0471	0.0158
	Socs2↑				

**Table 9:** The 84 genes differently expressed in ESG and normalized in HG (the *P* value and fold change of ESG versus CG ).

Gene ID	*P*	Fold change	Symbol	Description
287443	0.0414	2.0120	Acap1	ArfGAP with coiled-coil, ankyrin repeat, and PH domains 1
316628	0.0044	0.2748	Asb1	Ankyrin repeat and SOCS box-containing 1 (Asb1), mRNA
307970	0.0397	0.3289	Atxn1l	PREDICTED: similar to Ataxin-1 (Spinocerebellar ataxia type 1 protein homolog)
304127	0.0266	0.4310	Bach1	BTB and CNC homology 1, basic leucine zipper transcription factor 1
94342	0.0368	0.4621	Bat3	HLA-B-associated transcript 3, transcript variant 2,
308588	0.0241	0.4679	Car11	Carbonic anhydrase-related XI protein
81780	0.0349	2.6298	Ccl5	Chemokine (C-C motif) ligand 5
25405	0.0303	0.3845	Ccng1	Cyclin G1
362217	0.0393	0.4273	Cenpb	PREDICTED: centromere protein B
314004	0.0237	0.3330	Cmpk2	Cytidine monophosphate (UMP-CMP) kinase 2, mitochondrial, nuclear gene encoding mitochondrial protein
24273	0.0401	0.4750	Cryaa	Crystallin, alpha A
361729	0.0183	0.4488	Cybasc3	Cytochrome b, ascorbate dependent 3
308942	0.0369	0.3530	Dennd5a	DENN/MADD domain containing 5A
360583	0.0296	0.4192	Dhrs11	Dehydrogenase/reductase (SDR family) member 11
362293	0.0203	0.4955	Dnajb6	DnaJ (Hsp40) homolog, subfamily B, member 6
81655	0.0336	0.4654	Dync1li2	Dynein, cytoplasmic 1 light intermediate chain 2
59117	0.0343	0.3116	Eif2c2	Eukaryotic translation initiation factor 2C, 2
497983	0.0476	0.4848	Fam117a	Family with sequence similarity 117, member A
363083	0.0074	2.5213	Fbxl22	F-box and leucine-rich repeat protein 22
29292	0.0293	0.4455	Ftl	Ferritin, light polypeptide
54281	0.0281	0.3897	Furin	Furin (paired basic amino acid cleaving enzyme)
25172	0.0185	0.3991	Gata1	GATA binding protein 1
293267	0.0274	0.3516	Hbe1	Hemoglobin, epsilon 1
94164	0.0175	0.4161	Hbg1	Hemoglobin, gamma A
498008	0.0335	2.2484	Hexim1	Hexamethylene bis-acetamide inducible 1
365895	0.0417	0.3894	Hipk1	Homeodomain interacting protein kinase 1
288240	0.0022	0.3449	Hlcs	PREDICTED: holocarboxylase synthetase (biotin-(proprionyl-Coenzyme A-carboxylase (ATP-hydrolysing)) ligase)
293624	0.0080	0.3642	Irf7	Interferon regulatory factor 7
290985	0.0079	0.4919	Isca1	Iron-sulfur cluster assembly 1 homolog (S. cerevisiae)
298693	0.0462	0.3402	Isg15	ISG15 ubiquitin-like modifier
25118	0.0351	2.9262	Itga1	Integrin, alpha 1
300317	0.0493	0.4873	Kctd17	Potassium channel tetramerisation domain containing 17
25110	0.0410	2.6060	Klrd1	Killer cell lectin-like receptor, subfamily D, member 1
245955	0.0120	0.4700	Lgals3bp	Lectin, galactoside-binding, soluble, 3 binding protein
25476	0.0214	0.4406	Lgals9	Lectin, galactoside-binding, soluble, 9
100365370	0.0172	0.4588	LOC100365370	PREDICTED: nuclear LIM interactor-interacting factor 2-like
498145	0.0213	0.3006	LOC498145	Similar to RIKEN cDNA 2810453I06
679596	0.0155	0.4814	LOC679596	PREDICTED: similar to GABA(A) receptor-associated protein like 2
684112	0.0121	0.4067	LOC684112	PREDICTED: similar to KIAA0999 protein
293156	0.0090	0.4140	Lrtomt	Leucine rich transmembrane and 0-methyltransferase domain containing
294241	0.0443	0.2072	Ly6g6c	Lymphocyte antigen 6 complex, locus G6C
117558	0.0498	0.3267	Mylk2	Myosin light chain kinase 2
85482	0.0360	0.4205	Nbn	Nibrin
366998	0.0309	0.4486	Nfe2	Nuclear factor, erythroid derived 2
59115	0.0355	0.3302	Ninj2	Ninjurin 2
245980	0.0238	0.4878	Nr2f6	Nuclear receptor subfamily 2, group F, member 6
287328	0.0292	0.4931	Olr1439	Olfactory receptor 1439
287520	0.0498	0.4482	Olr1516	Olfactory receptor 1516
366104	0.0175	0.4251	Olr541	Olfactory receptor 541
246294	0.0120	0.3491	Optn	Optineurin
362973	0.0467	0.4896	Parvb	Parvin, beta
24649	0.0147	0.3899	Pim1	Pim-1 oncogene
64534	0.0423	2.1733	Pim3	Pim-3 oncogene
301173	0.0478	0.3759	Plcl2	Phospholipase C-like 2
310674	0.0473	0.4134	Plekho1	Pleckstrin homology domain containing, family O member 1
362873	0.0062	0.4404	Plxnc1	Plexin C1
362248	0.0215	0.4759	Procr	Protein C receptor, endothelial
309381	0.0286	2.2397	Pyroxd2	Pyridine nucleotide-disulphide oxidoreductase domain 2
171452	0.0460	0.3652	Rab3il1	RAB3A interacting protein
56820	0.0334	0.1273	Ramp3	Receptor (G protein-coupled) activity modifying protein 3
498659	0.0473	7.0377	RatNP-3b	Defensin RatNP-3 precursor
296408	0.0259	0.4348	RGD1311378	Similar to RIKEN cDNA 2010011I20
501644	0.0175	0.4259	RGD1561055	PREDICTED: similar to Ferritin light chain 2 (Ferritin L subunit 2) (Ferritin subunit LG)
65190	0.0454	0.3257	Rsad2	Radical S-adenosyl methionine domain containing 2
24974	0.0165	0.4619	RT1-A2	RT1 class Ia, locus A2 (RT1-A2)
414779	0.0105	0.4766	RT1-CE2	RT1 class I, locus CE2 (RT1-CE2)
266758	0.0163	2.6183	Sec11c	SEC11 homolog C (S. cerevisiae)
313057	0.0446	0.4886	Serinc2	Serine incorporator 2
498546	0.0120	0.1863	Serp2	Stress-associated endoplasmic reticulum protein family member 2
360636	0.0484	0.4722	Slc25a39	Solute carrier family 25, member 39 (Slc25a39)
192208	0.0472	0.3469	Slc38a5	Solute carrier family 38, member 5 (Slc38a5)
300191	0.0457	0.4485	Slc48a1	Solute carrier family 48 (heme transporter), member 1
64630	0.0330	0.4620	Snap23	Synaptosomal-associated protein 23
314251	0.0353	0.4407	Sptb	Spectrin, beta, erythrocytic
113894	0.0230	0.4367	Sqstm1	Sequestosome 1, transcript variant 1, mRNA
501146	0.0449	0.3749	Stradb	STE20-related kinase adaptor beta
24851	0.0449	0.3944	Tpm1	Tropomyosin 1, alpha
303167	0.0390	0.3720	Trim58	Predicted: tripartite motif-containing 58
362087	0.0450	0.3958	Ubac1	UBA domain containing 1
295704	0.0234	0.3510	Ube2l6	Ubiquitin-conjugating enzyme E2L 6
310633	0.0316	0.3751	Ubqln4	Ubiquilin 4
289229	0.0240	0.3468	Vangl2	Vang-like 2
24874	0.0262	2.6865	Vhl	Von Hippel-Lindau tumor suppressor
298765	0.0209	2.4995	Zfp36l2	Zinc finger protein 36, C3H type-like 2
